# Febuxostat, an Inhibitor of Xanthine Oxidase, Suppresses Lipopolysaccharide-Induced MCP-1 Production via MAPK Phosphatase-1-Mediated Inactivation of JNK

**DOI:** 10.1371/journal.pone.0075527

**Published:** 2013-09-25

**Authors:** Johji Nomura, Nathalie Busso, Annette Ives, Syunsuke Tsujimoto, Mizuho Tamura, Alexander So, Yoshihiro Yamanaka

**Affiliations:** 1 Pharmaceutical Department Research Laboratories, Teijin Institute for Bio-Medical Research, Teijin Pharma Limited, Hino, Tokyo, Japan; 2 Service of Rheumatology, Department of l’Appareil Locomoteur, Centre Hospitalier Universitaire Vaudois, University of Lausanne, Lausanne, Switzerland; University of California, Merced, United States of America

## Abstract

Excess reactive oxygen species (ROS) formation can trigger various pathological conditions such as inflammation, in which xanthine oxidase (XO) is one major enzymatic source of ROS. Although XO has been reported to play essential roles in inflammatory conditions, the molecular mechanisms underlying the involvement of XO in inflammatory pathways remain unclear. Febuxostat, a selective and potent inhibitor of XO, effectively inhibits not only the generation of uric acid but also the formation of ROS. In this study, therefore, we examined the effects of febuxostat on lipopolysaccharide (LPS)-mediated inflammatory responses. Here we show that febuxostat suppresses LPS-induced MCP-1 production and mRNA expression via activating MAPK phosphatase-1 (MKP-1) which, in turn, leads to dephosphorylation and inactivation of JNK in macrophages. Moreover, these effects of febuxostat are mediated by inhibiting XO-mediated intracellular ROS production. Taken together, our data suggest that XO mediates LPS-induced phosphorylation of JNK through ROS production and MKP-1 inactivation, leading to MCP-1 production in macrophages. These studies may bring new insights into the novel role of XO in regulating inflammatory process through MAPK phosphatase, and demonstrate the potential use of XO inhibitor in modulating the inflammatory processes.

## Introduction

Inflammation plays a fundamental role in a variety of chronic diseases such as atherosclerosis, rheumatoid arthritis, and chronic obstructive lung disease [1-3]. During inflammation, activated macrophages release pro-inflammatory cytokines that amplify cellular responses to injury as well as generating reactive oxygen species (ROS), which play an important role in the defense against invading organisms [4]. However, excess ROS production by activated cells can also provoke inflammation and tissue damage.

Xanthine oxidase (XO) is one of the major enzymatic sources of ROS. It is derived from xanthine dehydrogenase (XDH) either by proteolytic modification or reversible sulfhydryl oxidation [5]. XO catalyzes the oxidation of purine substrates, such as xanthine and hypoxanthine, producing uric acid and ROS [5]. XO has been reported to be up-regulated by various inflammatory stimuli such as LPS, hypoxia, and cytokines [6-9]. Augmented XO eventually causes excess ROS formation, leading to tissue damage. Pharmacological inhibitors of XO, such as febuxostat, allopurinol and oxypurinol have been reported to have an anti-inflammatory effect in various diseases such as atherosclerosis, chronic heart failure, acute lung injury, renal interstitial fibrosis and ischemic-reperfusion injury [10-15]. Thus, these findings demonstrate the essential role of XO in inflammatory conditions. However the mechanisms that link XO production to inflammation are not well understood.

We have studied the effects of modulating XO activity by LPS, in particular its effects on MCP-1, a potent chemotactic factor for monocytes and dendritic cells, and investigated if inhibition of XO leads to an altered inflammatory response. LPS is a main component of the outer membrane of Gram-negative bacteria, and previous studies have demonstrated that LPS up-regulates XO expression and activity. Pharmacological inhibition of XO protects against LPS-induced tissue injury [6,7]. Febuxostat, is a potent and selective XO inhibitor that effectively inhibits the formation of uric acid [16]. In this study, we found that febuxostat significantly suppresses LPS-induced MCP-1 production in macrophages and mice. These inhibitory effects were mediated by decreasing ROS formation and activating MKP-1, which leads to dephosphorylation and inactivation of JNK. These results may bring new insights into the novel role of XO in regulating inflammatory process through MAPK phosphatase.

## Materials and Methods

### Cell Culture

Human monocytic leukemia cell line THP-1 (TIB-202) was obtained from American Type Culture Collection and maintained in RPMI1640 (Life Technologies, Carlsbad, CA) supplemented with 10% FBS (Thermo Fisher Scientific Inc., Waltham, MA), 100 U/mL penicillin, and 100 mg/mL streptomycin (Life Technologies). To induce the differentiation into macrophage phenotype, cells were cultured for 72 h in RPMI1640 supplemented with 200 nM phorbol 12-myristate 13-acetate (PMA; Sigma-Aldrich, St. Louis, MO), 1% FBS, 100 U/mL penicillin, and 100 mg/mL streptomycin. For human primary monocyte-derived macrophages, CD14-positive monocytes from peripheral blood were purchased from PromoCell (Heidelberg, Germany). To induced the differentiation into M1 or M2 macrophages, monocytes were cultured for 7 days in RPMI-1640 supplemented with 50 ng/mL recombinant GM-CSF or M-CSF, respectively (PromoCell), 10% FBS, 100 U/mL penicillin, and 100 mg/mL streptomycin. Differentiated macrophages were pretreated for 10 min with the indicated concentration of febuxostat, allopurinol, oxypurinol or N-acetyl-L-cysteine (NAC; Sigma-Aldrich), and then stimulated with 100 ng/mL LPS from *Escherichia coli* (serotype O111:B4, Sigma-Aldrich). Cell viability was measured using the WST-8 cell counting kit (DOJINDO, Japan) according to manufacturer’s instructions.

### In vivo experiments

Female C57BL/6 mice (from Charles River) between 8-12 weeks of age were used for experiments. Animal experiments were performed in strict accordance to the Swiss Federal Regulations. The protocol was approved by the “Service de la consommation et des affaires vétérinaires du Canton de Vaud”, Switzerland. All efforts were made to minimize suffering and minimize the number of mice needed to assess statistical significance and experimental reproducibility. Mice were intraperitoneally treated with 0.5 ml of febuxostat at 1 mM or an equal volume of vehicle (0.6% DMSO) 30 min before an intra-peritoneal dose of 300 µg LPS (*Escherichia coli* O111:B4) in 0.5 ml sterile phosphate buffered saline (PBS). In addition control mice injected with vehicle or febuxostat alone were performed. After 6 hours, blood was collected and mice were euthanized by CO_2_ administration and peritoneal exudate cells were subsequently harvested by performing lavage with 3 ml of PBS. Lavage fluids were centrifuged at 450*g* for 10 min. Supernatants of lavage fluids and sera were used for MCP-1 ELISA measurement.

### Quantitative real-time RT-PCR (qRT-PCR)

Total RNA was extracted using TRIzol reagent (Life Technologies) according to the manufacturer’s instructions, and converted to cDNA using SuperScript VILO MasterMix (Life Technologies). The cDNA was amplified using SYBR^®^ Green (Life Technologies) with gene-specific primers on ABI PRISM 7500 system (Life Technologies). The oligonucleotide primers were: human MCP-1, 5’-CTCAGCCAGATGCAATCAAT-3’ and 5’-TCCTGAACCCACTTCTGCTT-3’; human TNF-α, 5’-CGAACATCCAACCTTCCCAA-3’ and CCCCAATTCTCTTTTTGAGCC-3’; human XDH/XO, 5’-GTGGATGCTGTGGAGGAGAT-3’ and 5’-TGCTTCCGAGGAGTGTCTTT-3’; human 18S rRNA, 5’-CGGCTACCACATCCAAGGAA-3’ and 5’-GCTGGAATTACCGCGGCT-3’. For data normalization, an endogenous control (18S rRNA) was determined for controlling the cDNA input and the relative units were calculated by a comparative *C*t method.

### Knockdown of XO gene with siRNA

THP-1 cells were treated for 48 h with 200 nM PMA, and transfected with 50 and 100 nM of control siRNA or human XO siRNA using DharmaFECT 2 (Thermo Fisher Scientific Inc.). Then, cells were treated with 100 ng/mL LPS for 20 h. On-TARGET plus SMART pool siRNA against human XO and non-targeting control siRNA were purchased from Thermo Fisher Scientific Inc.. Four target sequences in human XO are 5’-AGAGUGAGGUUGACAAGUU-3’, 5’-GGAGUAACAUAACUGGAAU-3’, 5’-UAGAGGAGCUACUAUUC-3’ and 5’-ACACGGAGAUUGGCAUUGA-3’.

### ELISA

The concentration of MCP-1 protein in culture media was measured with human MCP-1 DuoSet ELISA kit (R&D systems, Minneapolis, MN). The concentrations of MCP-1 protein in peritoneal lavage and serum were measured with mouse MCP-1 (eBioscience, San Diego, CA).

### Western blotting

Whole cell lysate was prepared and equal amount of lysate was loaded into SDS-PAGE. Western blotting was carried out using antibodies against phospho-JNK (81E11, Cell Signaling Technology, Beverly, MA), phospho-p38 (D3F9, Cell Signaling Technology), total IκB-α (L35A5, Cell Signaling Technology), and β-actin (AC-15, Sigma-Aldrich).

### Measurement of intracellular ROS level

Differentiated THP-1 cells were loaded for 30 min with 5 µM H_2_DCFDA (Life Technologies), and washed twice with HEPES-buffered saline (Life Technologies). Then, cells were pretreated for 10 min with the indicated concentration of febuxostat, allopurinol, oxypurinol or NAC, and stimulated with 100 ng/mL LPS in HBSS. Fluorescent intensity on 488 nm (excitation) and 530 nm (emission) was measured with Paradigm fluorescent plate reader (Molecular devices, Silicon Valley, CA).

### Measurement of ROS level produced by XO/xanthine

Buttermilk XO (Merck Millipore, Billerica, MA) was incubated for 60 min with 100 µM of xanthine, and ROS levels in the solutions were measured using the DCF indicator of Oxiselect^™^ in vitro ROS/RNS assay kit (Cell Biolabs, Inc., San Diego, CA) according to manufacturer’s instructions.

### Measurement of XO activity

Cells were homogenized with 50 mM phosphate buffer containing protease inhibitor cocktail, and sonicated for 10 min. The homogenate was incubated with 5 µM of pterine (Sigma-Aldrich) for 60 min, and fluorescent intensity on 360 nm (excitation) and 400 nm (emission) was measured with Paradigm fluorescent plate reader.

### Measurement of MKP-1 activity

Cell lysates were immunoprecipitated with anti-MKP-1 antibody (C-19, Santa Cruz Biotechnology, Inc., Santa Cruz, CA), and the phosphatase activities in the immunoprecipitates were determined with 6,8-fifluoro-4-methylumbelliferyl phosphate (Life Technologies). Fluorescent intensity on 360 nm (excitation) and 455 nm (emission) was measured with Paradigm fluorescent plate reader.

### Statistical analysis

All data are expressed as mean±SEM. For two-group comparisons, student’s *t* test was used. For multiple comparisons, one-way ANOVA followed by a Dunnett’s test was used to compare each group versus a vehicle-treated group. In time-dependent experiments, two-way ANOVA was used for two-group comparisons and Bonferroni test was used for comparisons at each time point. All data were statistically analyzed using GraphPad PRISM software version 4.01 (GraphPad, La Jolla, CA). Differences with a probability value of <0.05 were considered significant.

## Results

### Febuxostat inhibits LPS-induced XO activity and intracellular ROS formation in PMA-treated THP-1 cells

**Figure 1:**
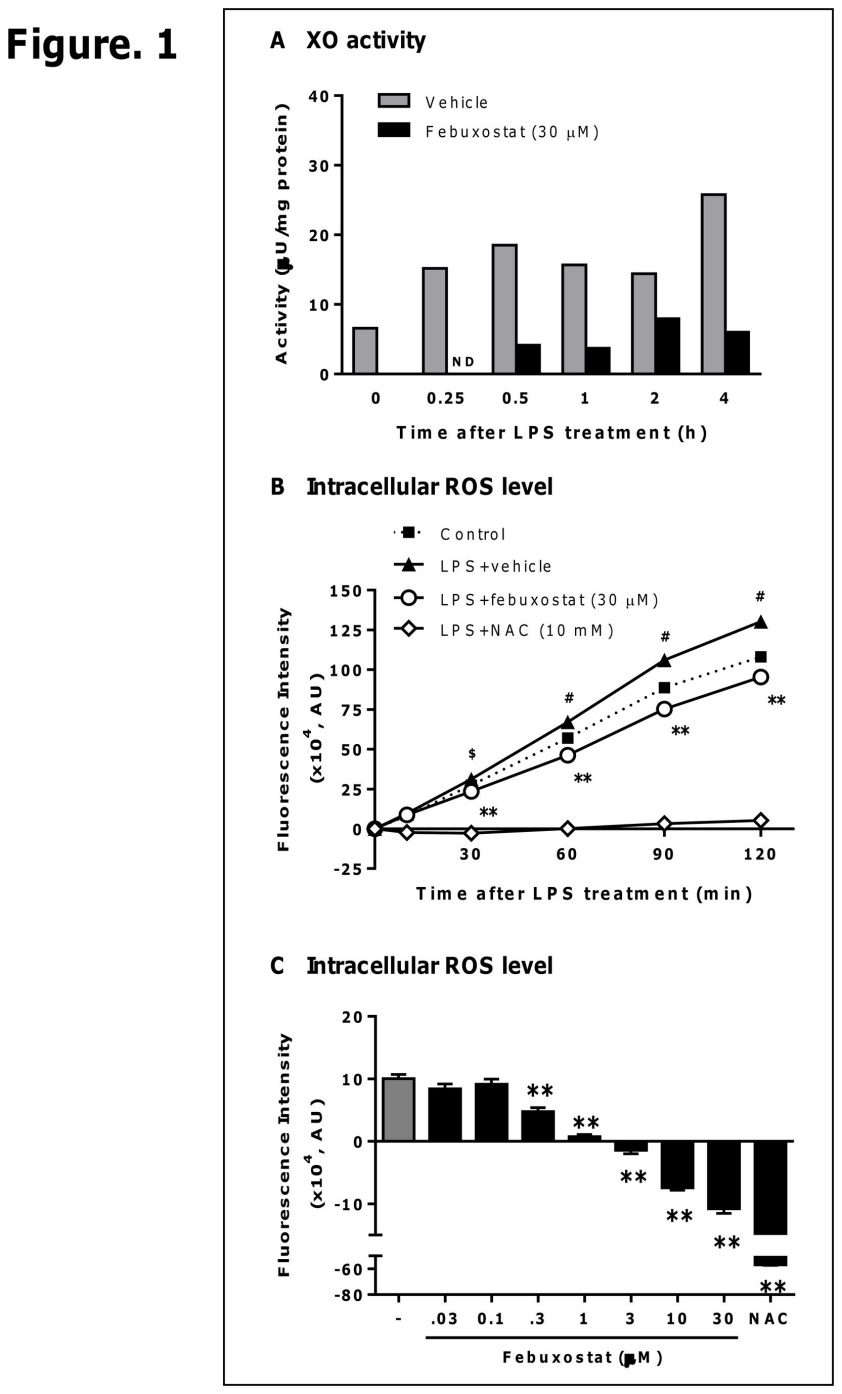
Febuxostat inhibits LPS-induced XO activity and intracellular ROS formation in PMA-treated THP-1 cells. (A) PMA-treated THP-1 cells were stimulated for the indicated time with 100 ng/mL LPS in the absence or presence of 30 µM febuxostat. The cell homogenates were incubated with 5 µM pterine for 60 min, and fluorescence intensity was measured. Data are shown as mean (n=3). (B) PMA-treated THP-1 cells were loaded for 30 min with 5 µM H _2_DCFDA, and then stimulated for the indicated times with 100 ng/mL LPS in the absence or presence of febuxostat (30 µM) or NAC (10 mM). Fluorescence intensity was measured. Data of one representative experiment (out of three experiments) are shown as mean±SEM (n=4). ^$^ *P*<0.01, ^#^ *P*<0.001 versus control-group, ^**^ *P*<0.001 versus vehicle/LPS-treated group. (C) Cells were loaded for 30 min with 5 µM H_2_DCFDA, and then stimulated for 60 min with 100 ng/mL LPS in the absence or presence of febuxostat (0.03, 0.1, 0.3, 1, 3, 10 and 30 µM) or NAC (10 mM). Fluorescence was measured and expressed as fluorescence intensity over LPS-untreated group. Data of one representative (out of three experiments) are shown as mean±SEM (n=4). ^#^ *P*<0.001 versus control-group, ^**^ *P*<0.01 versus vehicle/LPS-treated group.

In this study, we first examined whether LPS enhanced XO activity and whether febuxostat inhibits LPS-induced XO activity and intracellular ROS formation. PMA-treated THP-1 cells were stimulated with 100 ng/mL LPS in the presence or absence of febuxostat. As shown in Figure 1A, LPS treatment resulted in the rapid and sustained increase in XO activity 0.25-4 h after treatment. Febuxostat effectively inhibited basal and LPS-induced XO activity (Figure 1A). In addition, the analysis using H_2_DCFDA, an indicator of ROS, showed that intracellular ROS formation was significantly increased by LPS treatment in a time-dependent manner (Figure 1B). Febuxostat inhibited LPS-induced intracellular ROS formation whereas N-acetyl-L-cysteine (NAC), an antioxidant, deleted intracellular ROS completely (Figure 1B and 1C). These data demonstrated that febuxostat inhibits LPS-induced XO activity and XO-derived intracellular ROS formation.

### Febuxostat suppresses LPS-induced MCP-1 production by inhibiting XO-derived ROS formation in macrophages and mice

**Figure 2:**
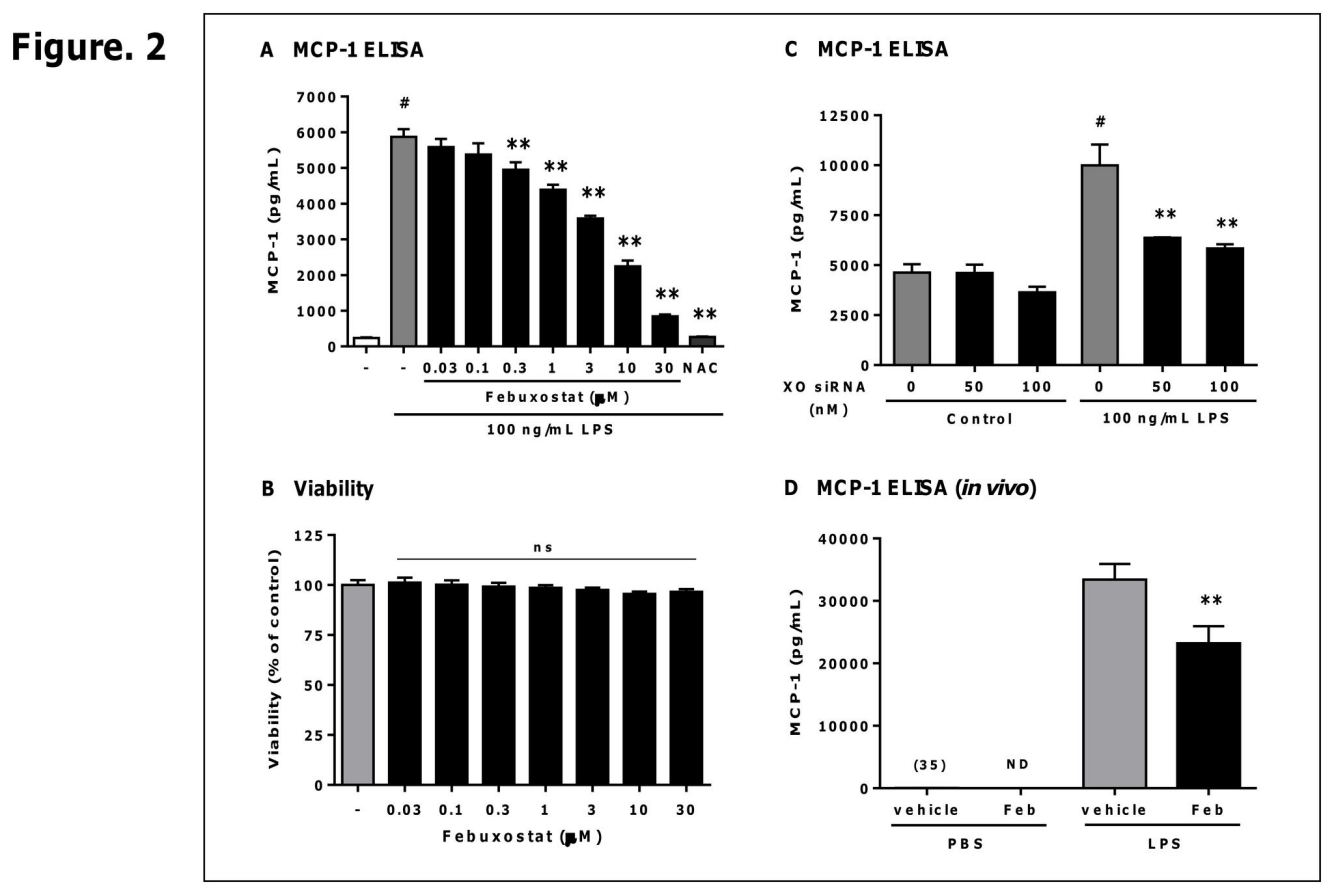
Febuxostat suppresses LPS-induced MCP-1 production by inhibiting XO-derived ROS formation in macrophages and mice. PMA-treated THP-1 cells were incubated for 20 h with 100 ng/mL LPS in the absence or presence of febuxostat or NAC. (A) The levels of MCP-1 in the supernatants were measured by ELISA. Data are shown as mean±SEM (n=4) of four independent experiments performed. ^#^ *P*<0.001 versus control-group, ^**^ *P*<0.01 versus vehicle/LPS-treated group. (B) Cytotoxicity was determined using WST-8. Data are shown as mean±SEM (n=3) of three independent experiments performed. ns, not significant versus vehicle/LPS-treated group. (C) PMA-treated THP-1 cells were transfected with 50 or 100 nM of XO siRNA or control siRNA, and then incubated for 20 h with 100 ng/mL LPS. The levels of MCP-1 in the supernatants were measured by ELISA. Data of one representative experiment (out of two experiments) are shown as mean±SEM (n=3). ^#^ *P*<0.01 versus 0 nM XO siRNA/control-group, ^**^ *P*<0.01 versus 0 nM XO siRNA/LPS-treated group. (D) Mice were intraperitoneally treated with 0.5 mL of vehicle (0.6% DMSO) or febuxostat (1 mM), and then injected with 300 µg of LPS. After 6 h, peritoneal lavage and serum were collected. The levels of MCP-1 in peritoneal lavage and serum were measured by ELISA. Data are shown as mean±SEM. LPS-treated mice with vehicle: n=20, with febuxostat: n=18, control mice with vehicle: n=4, and febuxostat: n=4. Value in parentheses indicates mean of MCP-1 concentration. ^**^ *P*<0.01 versus vehicle/LPS-treated group.

To determine the effects of febuxostat on LPS-induced inflammatory responses, we examined whether febuxostat suppresses LPS-induced MCP-1 production in macrophages. PMA-treated THP-1 cells were pretreated with 30 µM of febuxostat and then stimulated with 100 ng/mL LPS for 20 h. Although LPS treatment resulted in a large amount of MCP-1 in the supernatant, febuxostat significantly and dose-dependently inhibited LPS-induced production of MCP-1 without any effect on cytotoxicity (Figure 2A and 2B). NAC also inhibited LPS-induced production of MCP-1. Additionally, in human primary monocyte-derived M1 and M2 macrophages, febuxostat also suppressed MCP-1 production induced by LPS (Figure S1 in File S1). To confirm the inhibitory effect of febuxostat was dependent on the inhibition of XO, we tested whether XO siRNA, as well as febuxostat, suppressed LPS-induced MCP-1 production. XO knockdown mediated by XO siRNA but not control siRNA resulted in the inhibition of LPS-induced MCP-1 production (Figure 2C and S2 in File S1). Taken together, these results demonstrated that febuxostat suppresses LPS-induced MCP-1 production through inhibiting XO-derived ROS formation in macrophages.

Furthermore, we examined whether febuxostat also inhibits LPS-induced MCP-1 secretion in a *in vivo* model. Mice were intraperitoneally treated with febuxostat, then after 30 min mice were injected with 300 µg of LPS. In vehicle-treated mice, robust increases in MCP-1 levels were observed in peritoneal lavage whereas MCP-1 levels in febuxostat-treated mice were significantly reduced compared to vehicle-treated mice (Figure 2D). Similar data were obtained in serum from vehicle- and febuxostat-treated mice (vehicle: 98001±8624, febuxostat: 72545±7279, p<0.05). These data demonstrated that febuxostat inhibits LPS-induced MCP-1 production not only in a *in vitro* cell system but also in a *in vivo* mice model.

### Febuxostat suppresses LPS-induced MCP-1 mRNA expression without affecting mRNA stability

**Figure 3:**
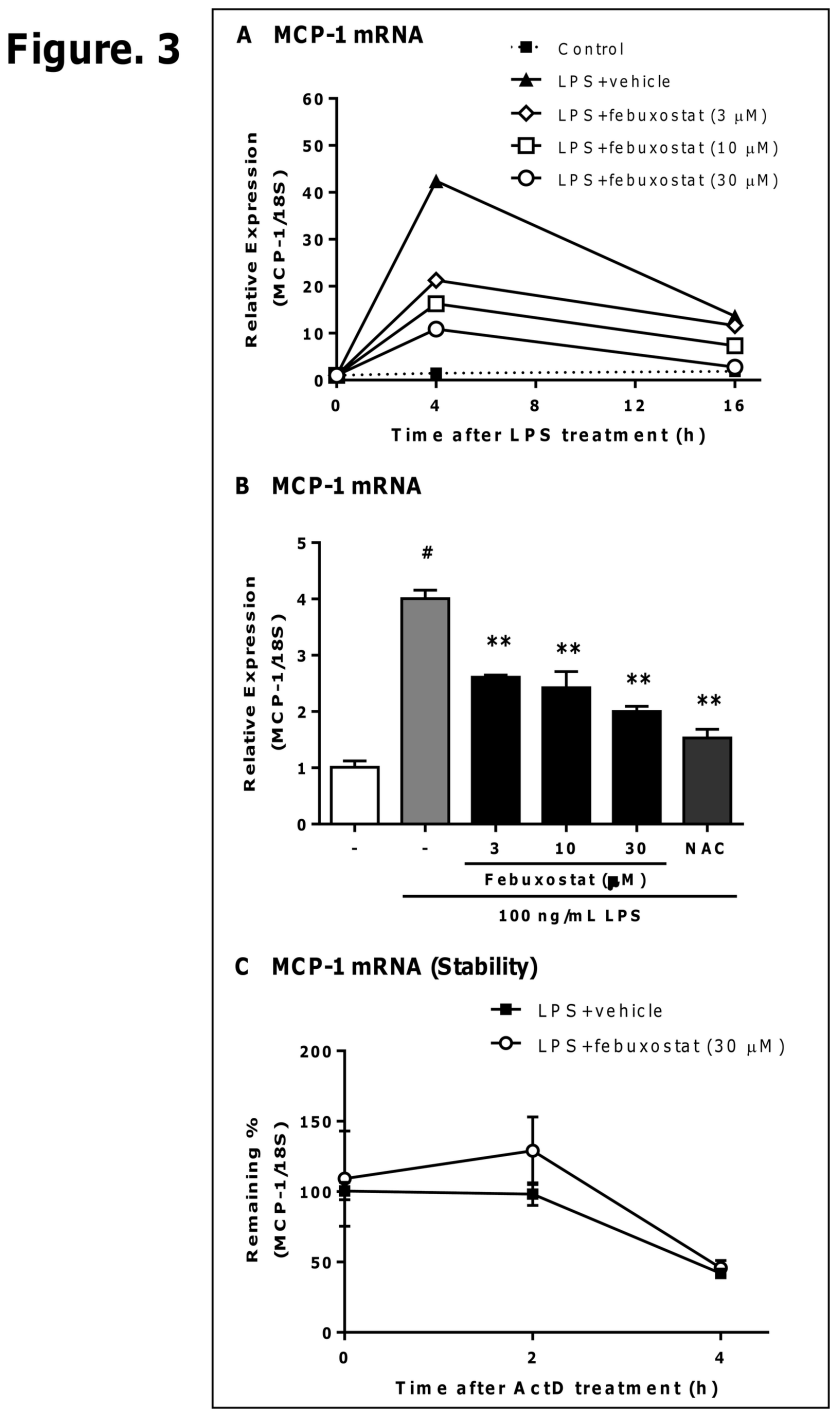
Febuxostat suppresses LPS-induced MCP-1 mRNA expression without affecting mRNA stability. (A) PMA-treated THP-1 cells were stimulated with 100 ng/mL of LPS for 0, 4 and 16 h in the absence or presence of febuxostat (3, 10 and 30 µM) or NAC (10 mM). Total RNA was extracted, and qRT-PCR was performed as described in materials and methods. Data are shown as mean of two independent experiments in which similar results were obtained. (B) Cells were stimulated with 100 ng/mL of LPS for 2 h in the absence or presence of febuxostat (3, 10 and 30 µM) or NAC (10 mM). Total RNA was extracted, and qRT-PCR was performed as described in materials and methods. Data are shown as mean±SEM (n=3) of three independent experiments. ^#^ *P*<0.001 versus control-group, ^**^ *P*<0.01 versus vehicle/LPS-treated group. (C) PMA-treated THP-1 cells were stimulated for 4 h with 100 ng/mL LPS in the absence or presence of febuxostat (30 µM), and then treated with 5 µg/mL Actinomycin D (ActD). Two or 4 h after ActD treatment, total RNA was extracted, and qRT-PCR was performed. Data are shown as mean±SEM (n=3) of three independent experiments.

Next, we examined whether febuxostat inhibits LPS-induced MCP-1 mRNA expression in PMA-treated THP-1 cells. LPS treatment results in the increase in MCP-1 mRNA expression in a time-dependent manner whereas febuxostat suppressed LPS-induced MCP-1 mRNA expression (Figure 3A). Consistent with the effects on protein level in Figure 2A, febuxostat significantly inhibited the induction of MCP-1 mRNA by LPS in a dose-dependent manner (Figure 3B). In addition, LPS-induced TNF-α expression was also inhibited by febuxostat (Figure S3 in File S1). Inhibitory effects of febuxostat were observed 2 h after LPS treatment, suggesting that febuxostat affected the transcription of MCP-1 mRNA. We next tested whether febuxostat affected the stability of MCP-1 mRNA. As shown in Figure 3C, febuxostat failed to affect the stability of MCP-1 mRNA. These results suggest that febuxostat inhibits LPS-induced MCP-1 mRNA expression without affecting mRNA stability, and regulates the transcription of MCP-1 by LPS.

### Febuxostat suppresses LPS-induced ROS formation and MCP-1 production more effectively than allopurinol and oxypurinol

**Figure 4:**
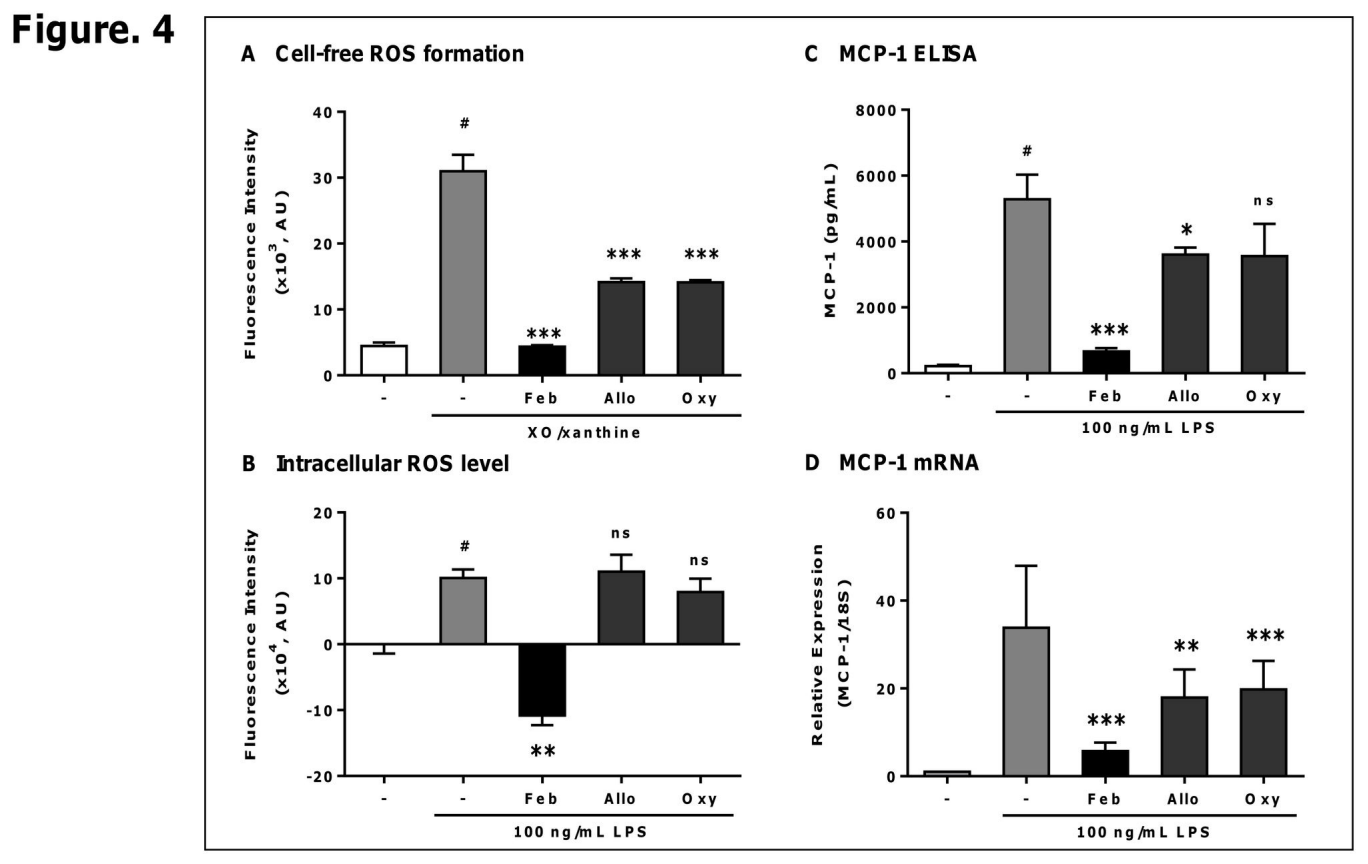
Febuxostat suppresses LPS-induced ROS formation and MCP-1 production more effectively than allopurinol and oxypurinol. (A) ROS was produced by buttermilk XO (10 mU/mL) and xanthine (100 µM) in the absence or presence of febuxostat (Feb, 100 nM), allopurinol (Allo, 100 µM) or oxypurinol (Oxy, 100 µM), and detected by DCF ROS indicator. Fluorescence was measured. Data are shown as mean±SEM (n=3) of three independent experiments. ^#^ *P*<0.0001 versus control-group,^***^ *P*<0.001 versus vehicle-treated group. (B) PMA-treated THP-1 cells were loaded for 30 min with 5 µM H_2_DCFDA, and then stimulated for 60 min with 100 ng/mL of LPS in the absence or presence of febuxostat (30 µM), allopurinol (300 µM) oroxypurinol (300 µM). Fluorescence was measured and expressed as fluorescence intensity over LPS-untreated group. Data of one representative experiment (out of three experiments) are shown as means ± SEM (n=4). ^#^ *P*<0.001 versus control-group, ^**^ *P*<0.01 versus vehicle/LPS-treated group. (C) PMA-treated THP-1 cells were incubated for 20 h with 100 ng/mL of LPS in the absence or presence of febuxostat (30 µM), allopurinol (300 µM) or oxypurinol (300 µM). The levels of MCP-1 in the supernatants were measured by ELISA. Data of one representative experiment (out of three experiments) are shown as mean±SEM (n=3). ^#^ *P*<0.001 versus control-group, ^*^ *P*<0.05, ^***^ *P*<0.001 versus vehicle/LPS-treated group. (D) Cells were stimulated for 16 h with 100 ng/mL of LPS in the absence or presence of febuxostat (30 µM), allopurinol (300 µM) or oxypurinol (300 µM). Total RNA was extracted, and qRT-PCR was performed as described in materials and methods. Data are shown as mean±SEM (n=3) of three independent experiments. ^**^ *P*<0.01, ^***^ *P*<0.001 versus vehicle/LPS-treated group.

It has been reported that other XOR inhibitors such as allopurinol and oxypurinol suppress intracellular ROS formation by XO, but their inhibitory effects are less potent than that of febuxostat [17]. Febuxostat (100 nM) potently inhibited ROS production by cell free XO/xanthine system, whereas the inhibitory effects of allopurinol or oxypurinol were partial, even at 1000-fold concentrations of febuxostat (Figure 4A). Similarly, in PMA-treated THP-1 cells allopurinol or oxypurinol had very weak or no inhibitory effects on LPS-induced intracellular ROS (Figure 4B). Consistent with these results, allopurinol or oxypurinol did not inhibit LPS-induced MCP-1 production and mRNA as efficiently as febuxostat (Figure 4C and 4D). These results indicated that febuxostat inhibits LPS-induced ROS formation and MCP-1 production more effectively than allopurinol and oxypurinol.

### Febuxostat suppresses LPS-induced activation of JNK

**Figure 5:**
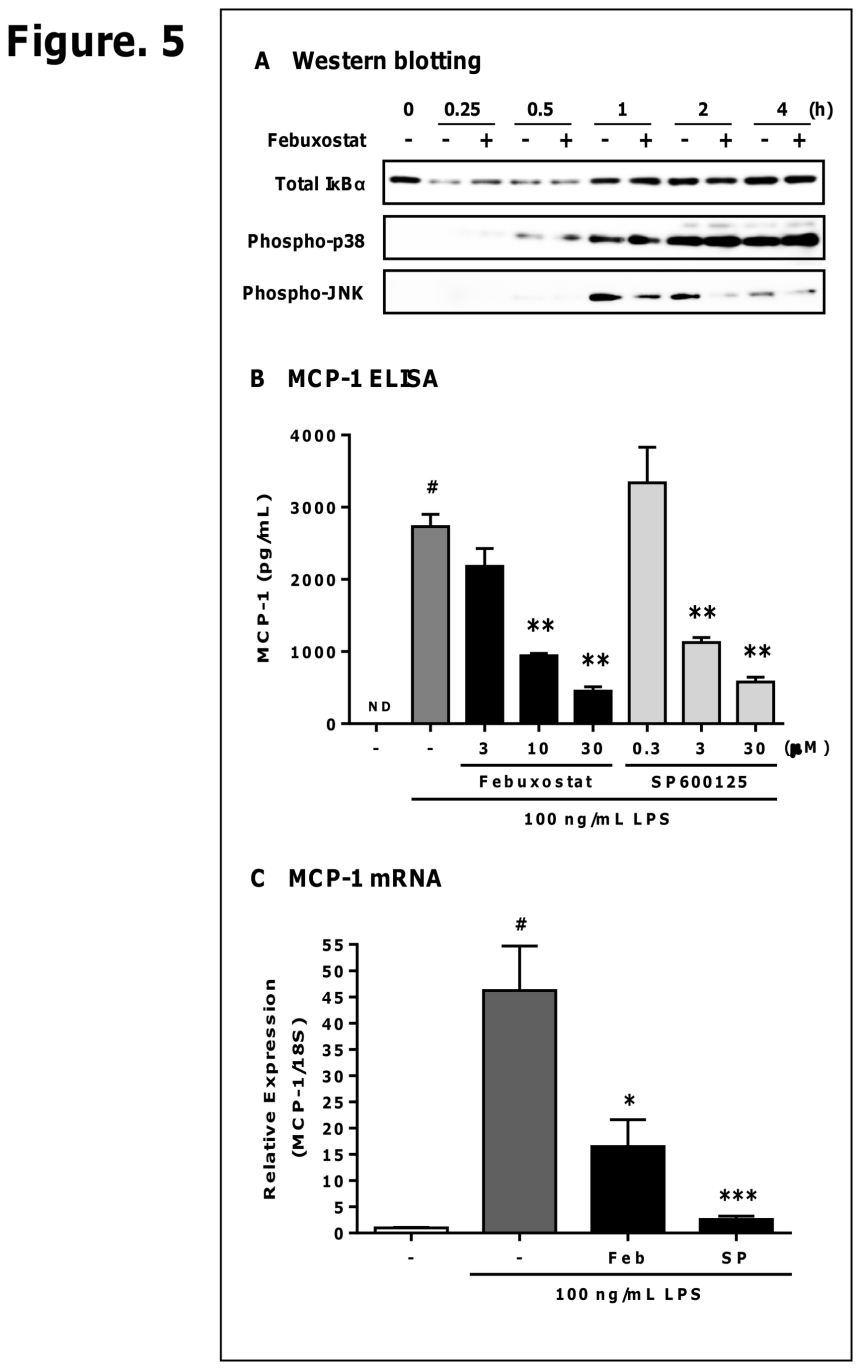
Febuxostat suppresses LPS-induced activation of JNK. Cells were stimulated with 100 ng/mL LPS in the presence or absence of febuxostat (30 µM). (A) Western blot analysis using anti-total IκBα, phospho-p38 and phospho-JNK antibodies. Equal amounts of proteins were loaded. The results are representative of two independent experiments in which similar results were obtained. (B) PMA-treated THP-1 cells were stimulated for 20 h with 100 ng/mL LPS in the presence or absence of SP600125 (0.3, 3 and 30 µM). The levels of MCP-1 in the supernatants were measured by ELISA. Data of one representative (out of three experiments) are shown as mean±SEM (n=3). ^#^ *P*<0.001 versus control-group, ^**^ *P*<0.01 versus vehicle/LPS-treated group. (C) Total RNA was extracted, and qRT-PCR was performed as described in materials and methods. Data are shown as mean±SEM (n=3) of three independent experiments. ^#^ *P*<0.001 versus control-group, ^*^ *P*<0.05, ^***^ *P*<0.001 versus vehicle/LPS-treated group.

In LPS signaling, activation of transcription factor NF-κB and MAPKs play essential roles in transcriptional induction of genes involved in inflammation, such as iNOS, COX-2 and TNF-α [18]. We therefore sought to investigate whether febuxostat inhibits signaling pathways, which play important roles in LPS-mediated MCP-1 induction in macrophages. As shown in Figure 5A, LPS transiently decreased total IκB-α level, showing the activation of NF-κB. However, pretreatment with febuxostat failed to change total IκB-α level. In addition, LPS treatment resulted in a time-dependent phosphorylation of JNK and p38 (Figure 5A), but not of ERK and MEK1 (data not shown). Febuxostat suppressed LPS-induced phosphorylation of JNK but not of p38. To determine the involvement of JNK in LPS-induced MCP-1, we examined the effects of LPS on MCP-1 production and mRNA expression in the absence or presence of SP600125, which is the specific inhibitor of JNK. As shown in Figure 5B and 5C, SP600125 suppressed LPS-induced MCP-1 production as well as mRNA expression, indicating that JNK plays essential role in LPS-induced MCP-1 production. These results suggest that febuxostat suppresses LPS-mediated activation of JNK.

### Febuxostat suppresses LPS-induced MCP-1 production via MKP-1-dependent inhibition of JNK through increasing MKP-1 activity

**Figure 6:**
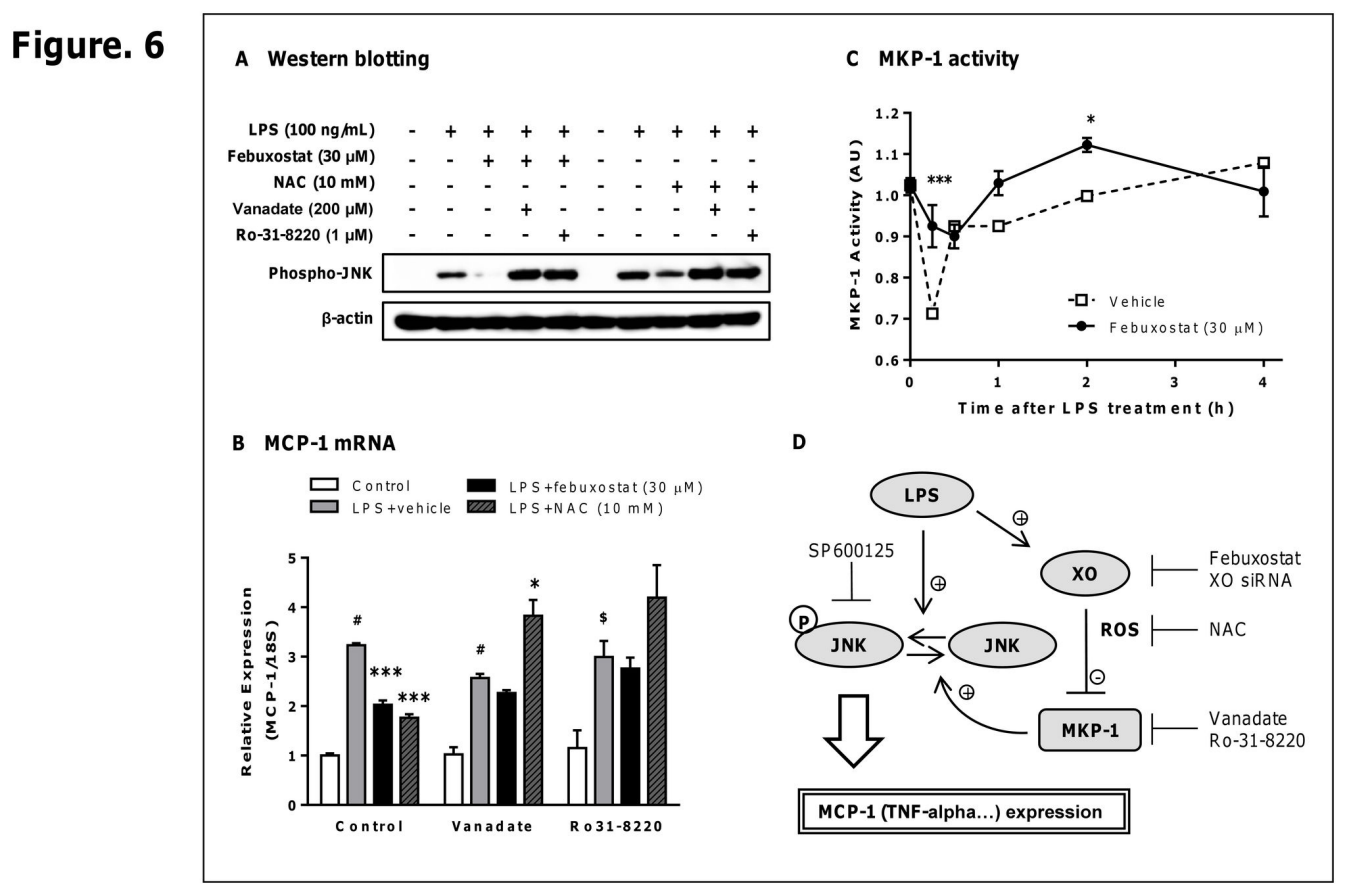
Febuxostat suppresses LPS-induced MCP-1 production via MKP-1-dependent inhibition of JNK through increasing MKP-1 activity. Cells incubated for 30 min with vanadate (200 µM) or Ro-31-8220 (1 µM) were pretreated for 10 min with febuxostat (30 µM) or NAC (10 mM), and then stimulated for 1 (A) or 2 h (B) with 100 ng/mL LPS. (A) Western blot analysis using anti-phospho-JNK and β-actin antibodies. Equal amounts of proteins were loaded. The results are representative of two independent experiments in which similar results were obtained. (B) Total RNA was extracted, and qRT-PCR was performed as described in materials and methods. Data are shown as mean±SEM (n=3) of three independent experiments. ^$^ *P*<0.01, ^#^ *P*<0.001 versus control-group, ^*^ *P*<0.05, ^***^ *P*<0.001 versus vehicle/LPS-treated group. (C) Cells were stimulated with 100 ng/mL of LPS in the presence or absence of febuxostat (30 µM). MKP-1 activities in immunoprecipitates were measured. Data are shown as mean±SEM (n=3) of three independent experiments. ^*^ *P*<0.05, ^***^ *P*<0.001 versus vehicle-treated group. (D) Schema of the signaling pathways involved in XO-mediated induction of MCP-1 expression by LPS. As indicated, LPS up-regulates MCP-1 expression via JNK-dependent pathway. XO activated by LPS causes decreased MKP-1 activity via accumulation of intracellular ROS that, in turn, leads to enhancement of JNK phosphorylation, the positive regulator of MCP-1 expression. Febuxostat suppresses LPS-induced MCP-1 expression through inhibiting XO/ROS/MKP-1/JNK pathway.

Because protein phosphatases have been shown to regulate the activation of JNK [19], we therefore examined whether protein phosphatases are involved in febuxostat-mediated inhibition of JNK phosphorylation. We first evaluated the effects of febuxostat on LPS-induced phosphorylation of JNK, and LPS-mediated MCP-1 expression in the presence or absence of protein phosphatase inhibitors. Here, we determined the effects of orthovanadate and Ro-31-8220, which are protein phosphatase inhibitor and MKP-1 inhibitor, respectively. As shown in Figure 6A, febuxostat-mediated inhibitions of phosphorylation of JNK were abolished by both orthovanadate and Ro-31-8220. Febuxostat no longer significantly inhibited LPS-induced MCP-1 expression in the presence of these inhibitors (Figure 6B). Moreover, the inhibitory effects of NAC were also abolished by these inhibitors. These data demonstrate that febuxostat inhibits LPS-induced MCP-1 expression via MKP-1-dependent inhibition of JNK activation.

Furthermore, we determined whether febuxostat affects the activity of MKP-1. After LPS treatment, MKP-1 activity was rapidly decreased, and followed by gradual increase (Figure 6C), suggesting that the change in phosphatase activity after LPS treatment was likely to affect the phosphorylation levels of JNK. Consistent with time points at which febuxostat-mediated inhibition of JNK activation was observed, febuxostat significantly increased phosphatase activity 1-2 hours after LPS treatment (Figure 6C). Taken together, these results suggested that febuxostat suppresses LPS-induced MCP-1 production via MKP-1-dependent inhibition of JNK through increasing MKP-1 activity.

## Discussion

Although several studies have reported that XO plays the fundamental roles in inflammation, the molecular mechanisms underlying the involvement of XO in inflammatory responses remain unclear. We here examined the effects of febuxostat, a selective and potent inhibitor of XO, on LPS-induced inflammatory signals. In this study, we found that febuxostat significantly suppresses LPS-induced MCP-1 production in human macrophages and *in vivo* in mice (Figure 2 and Figure S1 in File S1). The inhibitory effects of febuxostat on LPS-induced MCP-1 were mimicked by XO knockdown (Figure 2C), as well as the other XO inhibitors such as allopurinol and oxypurinol (Figure 4C). In addition, exogenous XO induced febuxostat-inhibitable MCP-1 production in PMA-treated THP-1 cells (Figure S4 in File S1). Taken together, these data suggested that XO plays essential roles in LPS-induced MCP-1 production, and that our *in vitro* system using PMA-treated THP-1 cells is well suitable for investigating the mechanisms underlying XO-mediated inflammatory pathways.

MKP-1, a protein phosphatase with dual specificity (Ser/Thr or Thr/Tyr), is responsible for the inactivation of JNK and p38, and therefore controls MAPK-dependent inflammation during the innate immune response [20,21]. Because MKP-1 activity has reported to be regulated by oxidative stress [22], we hypothesized that XO-derived ROS might contribute to decreased MKP-1 activity following LPS stimulation. Supporting this hypothesis, NAC-mediated inactivation of JNK was abolished by orthovanadate or Ro-31-8220 (Figure 6A), and decrease in MKP-1 activity by LPS was recovered by NAC (data not shown). Thus, LPS decreases MKP-1 activity via XO-derived ROS, leading to enhancement of JNK phosphorylation and ultimately to the induction of MCP-1. On the other hand, febuxostat inhibits XO-derived ROS and increases MKP-1 activity, which leads to dephosphorylation and inactivation of JNK. This is supported by the findings that febuxostat failed to inhibit LPS-induced MCP-1 production and phosphorylation of JNK in the presence of orthovanadate or Ro-31-8220 (Figure 6A and 6B). Additionally, febuxostat enhanced MKP-1 mRNA levels, whereas it did not alter the mRNA levels of the other MKPs, such as MKP-3 and -5 (data not shown). To our knowledge, this is the first report showing a link between XO and MKP-1, representing an important and new regulatory mechanism in inflammation.

XO is well known to produce not only ROS but also uric acid. Several studies have reported that uric acid induced MCP-1 production in tubular epithelial cells, adipocytes and vascular smooth muscle cells [23-25]. However, our data revealed that XO-derived ROS is involved in LPS-induced MCP-1 production in PMA-treated THP-1 cells. In fact, LPS enhanced XO activity and increased intracellular ROS formation (Figure 1). In addition, febuxostat as well as NAC attenuated increases in ROS formation and MCP-1 production (Figures 1 and 2). Furthermore, uric acid at concentrations equivalent to that found in hyperuricemic blood failed to affect basal and LPS-induced MCP-1 levels (Figure S5 in File S1). Taken together, these findings demonstrate the crucial role of ROS in LPS-induced MCP-1 production in macrophages.

Inhibitory effects of febuxostat on the expression of cytokines/chemokines were selective: febuxostat also inhibited LPS-induced TNF-α, VCAM-1 and MMP9 expression, as well as MCP-1, but did not affect IL-8 (Figure S3 in File S1 and data not shown). Additionally, inhibitory profiles of febuxostat did not completely correspond to that of SP600125 (data not shown), suggesting the possible involvement of signal molecules other than JNK in XO-mediated inflammatory responses. Further studies are required to clarify this possibility.

The inhibitory effect of febuxostat against LPS-induced MCP-1 production obtained in the human THP-1 cell line was reproduced in human primary macrophages. In both cell types, the IC50 dose was close to 10 µM. It is interesting to note that in clinical studies when febuxostat was given at 120 mg once daily for 13 days, the C_max_ of febuxostat in plasma is around 17 µM [26,27]. Thus, the inhibitory doses of febuxostat *in vitro* are in the same range of the doses found in plasma from febuxostat-treated patients, suggesting that the anti-inflammatory effects of febuxostat found *in vitro* may be relevant *in vivo*. Further clinical studies are needed to confirm the potential anti-inflammatory role of XO inhibiotrs.

In conclusion, febuxostat suppresses LPS-induced MCP-1 production and mRNA expression via activation of MKP-1 which, in turn, leads to dephosphorylation and inactivation of JNK in macrophages. These findings suggest that XO contributes to inflammatory responses via MKP-1-dependent JNK activation through intracellular ROS formation. These studies may bring new insights into a novel role of XO in regulating inflammatory process through MAPK phosphatase, and demonstrate the potential use of XO inhibitor in modulating the inflammatory processes.

## Supporting Information

File S1 **Supporting figures (Figures S1-S5).** **Figure S1**. Human primary monocyte-derived macrophages were differentiated from CD14-positive monocytes with 50 ng/mL GM-CSF or M-CSF for 7 days, then stimulated with 15.6 ng/mL LPS in the absence or presence of febuxostat. The levels of MCP-1 in the supernatants were measured by ELISA. Data are shown as mean of two independent experiments in which similar results were obtained. **Figure S2**. PMA-treated THP-1 cells were transfected with 50 or 100 nM of XO siRNA or control siRNA, and then incubated for 20 h with 100 ng/mL LPS. Total RNA was extracted from 3 wells, and qRT-PCR was performed as described in materials and methods. Data are shown as mean of two independent experiments in which similar results were obtained. **Figure S3**. PMA-treated THP-1 cells were stimulated with 100 ng/mL of LPS for 2 h in the absence or presence of febuxostat (3, 10 and 30 µM) or NAC (10 mM). Total RNA was extracted, and qRT-PCR was performed as described in materials and methods. Data are shown as mean±SEM (n=3) of three independent experiments. ^#^ *P*<0.001 versus control-group, ^*^ *P*<0.05, ^**^ *P*<0.01 versus vehicle/LPS-treated group. **Figure S4**. PMA-treated THP-1 cells were stimulated with 10 mU/mL of XO for 20 h in the absence or presence of febuxostat (10 µM). The levels of MCP-1 in the supernatants were measured by ELISA. Data are shown as mean of two independent experiments in which similar results were obtained. **Figure S5**. PMA-treated THP-1 cells were stimulated with 100 ng/mL of LPS for 20 h in the absence or presence of uric acid (0, 500 and 1000 µM). The levels of MCP-1 in the supernatants were measured by ELISA. Data are shown as mean±SEM (n=3).
(TIF)
